# Where Do We Stand?

**Published:** 1891-02-21

**Authors:** 


// The Hospital

A Weekly Journal of

Science, Medicine, Nursing, and Philanthropy.

*For Hospitals, Asylums, Infirmaries, Dispensaries, Medical Practitioners, Students, Nurses, and the Charitable Public.*

Vol. IX.—No. 230.] [February 21, 1891.

### Where Do We Stand?

According to the *Speaker*, Mr. John Morley was for fifteen years editor of the *Fortnightly Review*, and during that time he "kept a cockpit of his own." But the *Speaker* thinks that Mr. Morley's inner and most approved idea of a newspaper or magazine is not that of a "cockpit" where practically indiscriminate fighting is the order of the day, but that where an editor can see around him a faithful band of contributors who start together "from a common set of principles, accept a common programme of practical applications, and set to work in earnest, and with due order and distribution of parts, to advocate the common cause." This expresses very clearly and briefly what one conceives ought to be, and is, the line of things adopted by every honest newspaper. The first requisite for a newspaper is the same as the first requisite for a man, namely, that it have a purpose. It has been brought into existence to do something; and it ought to do that something with understanding, persistence, and might, if it be worth doing. He is a poor creature of a man who does not deserve a friend; and it is a very sorry cause which is not worthy of an advocate. Even the opponents of any given cause ought to be glad when it has found an advocate; because they will then see for themselves its strength as well as its weakness; its wisdom and justice, if it have any, as well as its foolishness and unworthiness. An honest newspaper should strive to be the advocate of a good cause.

The *raison d'être* of this journal has sometimes been called in question, and its position has been held to require definition. What is The Hospital: and where does it stand? It stands for Hospitals in the first place; but for Hospitals as representing the whole science and art of healing; and it stands, therefore, for all who are in any way concerned with the medical art; for patients, for physicians and surgeons, for nurses and midwives, and for every auxiliary of every kind which has to do with the sick and their care and cure. Hospitals in themselves are nothing apart from their patients and the attendants of their patients; and doctors and nurses are nothing apart from the great science and art of healing. In standing for Hospitals, The Hospital, in a word, stands for "medicine" and all that appertains thereunto.

But though we stand for medicine we do not "keep a cockpit of our own." The cockpit we believe and hope is a little out of date, at any rate among men of any culture. The average man may still love fighting, but he prefers the contests of the intellect, not the brutal vulgarities of the prize-ring. We fight in these pages a good deal; and we do not always fight with "gloves" on. But this much we can say with certainty, that if we sometimes have no gloves on our hands when we fight, we never have any malice in our hearts. It may occasionally appear to medical readers that we are taking the side of the public against them, and they may be angry with us accordingly. If ever we take the side of the public, or a portion of the public, as against the medical profession, or a portion of it, it is not because we are less loyal to medicine, but more loyal. He who aids a man or a body of men to do that which is wrong and injurious, is not a friend but an enemy to the very men he aids.

It has seemed desirable to devote some space to the definition of our position because of the attitude we have taken up with regard to the Midwives' Registration Bill. The medical profession has been divided into two opposing camps by the introduction of this measure. The leaders of both parties have sought our aid, and that during a considerable period of time. We have been anxious, if possible, to avoid taking a side. But as practical men know, there comes a time when a newspaper, as well as an individual must take a side, unless it would lose its just self-respect, and its title to be considered of any consequence and moment at all. In taking one side we do not thereby affirm that our party is absolutely and universally right, and that the opposite party is absolutely and universally wrong. In the present crisis we have simply done our best to judge fairly the facts and merits of the case, and we have decided that, in our judgment, the supporters and defenders of the education and registration of midwives have more general right and justice on their side than the opponents of such education and registration.

Stated calmly and dispassionately, that is our position. But then, having taken a side, what is the next thing to be done? We must fight. Now, fighting is not a matter of smiles and kid gloves; it is an affair of serious earnest. It must always, of course, be fair, strictly and honourably fair; but it must also be strenuous. If it be fighting at all, worthy fighting, it cannot but be strenuous. We think our side is right, and we fight to win. We think the other side is wrong, and we fight to beat it and drive it from the field. In this, surely, we may claim not only the respect, but also the sympathy of our opponents. They think they are right in fighting against us. Then let them do their utmost, and, as the warriors of old used to say, "God defend the right!"

It is not likely that any harm will come to any single member of the medical profession by the passing of the Midwives' Bill, much less by the mere discussion of it. But in order that no harm may arise, those who take part in the discussion must do so with reason and temper. The public are sensitively alive to the sayings and doings of doctors at the present moment. Whatever medical men may themselves think, there is not the slightest doubt that the world at large regards the medical profession from a purely utilitarian point of view. Doctors, they say, have certain social and State privileges. Do they prove by their worthiness and public utility that they deserve those privileges, or do they use their position to make claims of an arrogant, selfish, and impossible character? Do they, in return for their privileges, strive their utmost to ensure and promote measures of public utility? Or do they use their privileges as a Parisian street barricade, from behind which they can fire determined shots of a character dangerous to the public and the general well-being?

The opponents of the Registration Bill should take the measure of those medical men who support it. We do not wish to attach undue importance to names, but there are names in medicine whose owners have earned a right to attention. Many of the medical men who stand for the registration of midwives are men whose character and whose sympathy with all ranks of their professional brethren are absolutely above suspicion. If they thought the poorest of their fellow-doctors was going to be wronged by a single hair's-breadth they would fly to his rescue. Why do these medical men of statesmen-like intelligence and high moral worth stand for the education and and registration of midwives? Because they think that the public safety demands the enactment of such a measure, and that medicine itself will not be the loser but the gainer thereby. Against those who are opposing the measure we have nothing to say. So far as they are known to ourselves they are equal in personal and professional merit to the men of our own side. But we do not think that they are equal in experience and knowledge of the world. State legislation requires something more than good intentions and sound professional knowledge. That something, it appears to us, the opponents of midwives' registration are deficient in. They should, we think, reconsider their position, re-weigh the facts of the case, compare their own country's regulations with those of other civilised nations, and, putting aside all temporary arguments, should act upon the great and humane principle of doing as they would be done by, whatever be the consequences that may follow.

